# A Report of Three Interesting Cases

**Published:** 1902-07

**Authors:** George M. Boon

**Affiliations:** Southwick, Mass.


### REPORTS OF CASES.

### A REPORT OF THREE INTERESTING CASES.

By George M. Boon, V.S.,
SOUTHWICK, MASS.

The following three cases possess some features of interest which, I trust, will render them worthy of record and also serve to illustrate a method of treatment which I recently found of service:

Case I.—A black mare, aged eight years. Diagnosis: Opening of the humero-radial articulation near side,as the result of injury from a barb-wire fence. This mare had been under the care of another veterinarian, who was called in at the time of the accident, three weeks previously, and sewed up the wound, which was quite extensive and required some dozen or more sutures. The opening into the joint was not discovered at the time, and may not have been present, but may have supervened later from the effects of her scratching to relieve the itching caused by the cicatrization of the wound. At the time of my first visit the mare was in great agony, and the synovial fluid was escaping from the joint through a fistula about three-sixteenths of an inch in diameter. It was ejected in spurts every two or three minutes, or about twenty spurts to the minute, these being augmented by isochronous movements of the limb. I informed the owner that the prognosis was grave, and advised the use of protargol, a supply of which I had just received. I made up a solution of protargol, forty grains to one ounce of distilled water, and ordered him to make an injection into the joint three times daily. This he did for ten days, at the end of which time the fistula was entirely closed up, and to-day, June 7th, nearly eight months later, the animal is sound and well, and, with the exception of a small scar, no one would ever suspect that any such accident had happened.

Case II.—A gray mare,aged ten years, a good roadster, has had a bad fistula of the withers for two years. She had been operated upon quite a number of times by several well-known veterinarians; but a month or six weeks after healing the fistula would reopen. I was called to see her on November 7, 1901, and found her in great distress, somewhat off her feed, and the withers very badly swollen on each side to the size of a child's head. There was a tendency to pointing on the near side close to the top. I immediately opened the swelling at this point, which was followed by an evacuation of a surprisingly large amount of pus—I should say there was a quart of it, at least. I then made an exploration of the cavity with a soft metallic probe, finding among other conditions a necrosis of several of the spinous processes of the dorsal vertebra. A plug of caustic potash was then inserted through the opening, which was allowed to remain until my next visit. November 9th, after throwing the animal, I removed the necrosed spinous processes, and made a free opening at the bottom of the cavity. I inserted a rubber tube through it at the top, thus promoting free drainage. I then washed out the cavity with a solution of creolin (Pearson), one ounce to one pint of water, after which I injected protargol, 40 grains; glycerin, 2 drachms; water, q. s. 2 ounces.

November 10th the owner informed me that the mare had acted better since this morning than for some time past, not being in such agony, and had eaten her breakfast and dinner with a relish. I again washed out the cavity with a solution of creolin (Pearson), and injected protargol in the same way as I had before. The owner being afraid to treat her himself, as he had been badly kicked by her the last time she was being treated, he requested me to give the case my personal attention daily, which I agreed to do. November 11th, the mare has a good appetite, and the pains seem to be entirely absent; in fact, she let me make an examination of the fistula without putting on a twitch to control her, which had never been done any time since she was first affected. I again washed out the cavity with a solution of creolin, and injected protargol. November 12th, 13th, and 14th, same treatment. November 15th, swelling of the parts has quite perceptibly diminished; treatment continued. November 16th, 17th, and 18th, no change in treatment. November 19th, the discharge has somewhat diminished in quantity; the odor is less marked, and the granulations look more healthy; treatment as above. November 20th and 21st, treatment is continued. November 22d, very little pus, and no odor to it whatever, showing the necrosis to be practically arrested. I removed the drainage-tube and washed out the cavity with creolin solution and injected protargol. November 23d, 24th, and 25th, no change. November 26th, the cavity and openings are healing nicely; the pus appears healthy, and the mare seems better, according to the owner's statement, than she has been for months; same treatment.

November 27th, 28th, and 29th, conditions improving; treatment continued. November 30th, fistulous opening at the top is closing up nicely, and very little if any pus is escaping from the bottom opening; continued treatment. December 1st, upper opening nearly closed, and the cavity healing nicely; same treatment. December 2d, parts healing nicely. The superior opening has closed entirely, and the lower one almost completely. I now discontinued the creolin solution, and confined myself to injections of protargol. December 3d and 4th, no discharge; protargol injection. December 5th, I did not see her, being absent from town on legal business. December 6th, lower opening closed. I now gave the owner instructions to bathe the parts morning and evening with witch-hazel and arnica, equal parts, for several weeks. December 25th, the owner drove the mare the first time in four months. He called to see me. She is now in perfect health, and the owner informs me that she drives better than she has in two years. June 2d, the mare is sound, is being driven daily, and is entered in one of the road races which we have here on one of our highways semi-monthly.

Case III.—Bay gelding, aged nine years. March 28, 1902, diagnosis: Lacerated wounds of the cheek and parotid gland, with escape of salivary secretion and formation of pus, caused by a nail in the upper part of the manger, where he had climbed in trying to reach hay. Treatment: Sutures were inserted to hold the edges of the wound together, and cold-water irrigation employed, which was kept up until I saw him the next day. As the accident happened several days previously, I gave the owner to understand that a salivary fistula might be expected, as mastication produced quite an abundant flow of saliva over the sides of the cheek and gland. March 29th and 30th, cold-water irrigation continued. March 31st, I found abscess developing along the anterior border of the inferior portion of the parotid gland. I made a free opening of the lower extremity of the swelling, allowing the escape of pus, which discharged quite freely, and then washed out the pus cavity with a solution of creolin, 1 : 32, and injected protargol, 40 grains, to water, 1 ounce. I instructed the owner to do likewise twice daily until I called again. April 2d, the horse is doing nicely, and the swelling is almost gone; same treatment continued. April 4th, the owner informed me that I need not call again. May 5th, he again sent for me in haste to see the horse, and I found he had neglected him almost entirely. An ignorant quack of the worst kind had been called in to treat him about three days after my last visit. He informed the owner that by applying what he called a cast (a composition of wax, glue, and cow dung) over the opening that it would cease running and heal up at once. The result of this treatment was a quite large and diffused swelling of the side of the cheek and gland, which was filled with pus and saliva. I immediately shaved off this so-called cast, opened up the abscess at the old points, and inserted a rubber drainage-tube. I then washed out the cavity with a solution of creolin, 1 : 32, and injected protargol, 40 grains to 1 ounce of water, using about 2 drachms twice daily. This treatment I personally continued for one week, when I removed the drainage-tube, and continued the creolin solution and protargol injections for three days, after which the latter only were employed. At the end of a week the abscess was entirely healed and the fistulous tract completely obliterated, with no swelling or thickening of the gland or adjacent tissues. June 9th, having been called to see a cow at the same place, which had badly lacerated one of her teats with a barb-wire fence, I found that the horse at this time was perfectly well and that not even a scar remained to show that a fistula had existed. I would say in this connection that I also treated the cow with protargol, it being applied to the edges of the wound, which was coapted by three catgut sutures. I feel sure that under this treatment the wound will heal nicely, for the drug has acted almost as a specific in one or two simlar cases where the milk duct was badly cut and the secretion was escaping through the wound. July 21st, the last-mentioned case has entirely healed up, and except for marks of cicatrix no one would ever suspect anything having happened to her.

